# Perception of weather and seasonal drought forecasts and its impact on livelihood in East Nusa Tenggara, Indonesia

**DOI:** 10.1016/j.heliyon.2019.e02360

**Published:** 2019-08-30

**Authors:** Heri Kuswanto, Fausania Hibatullah, Eddy Setiaji Soedjono

**Affiliations:** a Department of Statistics, Institut Teknologi Sepuluh Nopember (ITS), Kampus ITS Sukolilo Surabaya, 60111, Indonesia; b Centre for Earth, Disaster and Climate Change, Institut Teknologi Sepuluh Nopember (ITS), Kampus ITS Suklilo Surabaya, 60111, Indonesia; c Department of Environmental Engineering, Institut Teknologi Sepuluh Nopember (ITS), Kampus ITS Sukolilo Surabaya, 60111, Indonesia

**Keywords:** Information science, Sociology, Forecast, Survey, Livelihood, Resilience

## Abstract

A cross-sectional study was conducted in 2018 to assess the perception of households on drought forecasts and its impact on crop and livestock losses. A total of 300 households from seven districts in East Nusa Tenggara Indonesia were considered. The study indicated that the majority of the households are poor families with low education background. They sold poultry for income generation during drought events. The survey revealed that only small percentage of the households usied forecast to support their livelihood management. The statistical test confirmed that the use of forecast did not necessarily impacted the crop loss. However, the crops were significantly affected by the response to drought forecast. Households that changed their agricultural practice experienced significantly different losses than households that did not do anything differently to their crops. The households argued that the accuracy of the forecasts issued by the government was very low. Therefore, it is recommended that policymakers and government authorities provide more accurate forecasts and a better strategy to increase household awareness of using drought forecasts.

## 1 Introduction

Drought has became a major threat in poor rural communities in developing countries. Numerous studies have found that drought frequency will increase and the magnitude of droughts will be greater in the future due to climate change. Indonesia is one of the most vulnerable countries in the world to climate change and prone to natural disasters. Based on statistics reported by the National Agency for Disaster Management (BNPB), within the last five years there has been a considerable number of natural disasters — i.e. about 1,811 events in 2012, 1,961 events in 2014 and 2,372 events in 2017 (BNPB, 2017). Flood, landslide, tornado, and drought were the top four disasters based on the frequency of the event. In terms of impact, drought was ranked as the second biggest disaster after flood, and the number of victims was about 100,000 people in 2018. Setiawan et al. (2017) discussed the spatio-temporal characteristics of Indonesian drought related to El Niño events and its predictability using the multi-model ensemble. They found that drought Indonesia is highly impacted by El-Nino, and the degree of severity is predicted to be higher in the feature. This finding is consistent with the Work of Kuswanto et al. (2018) who found that the drought magnitude and duration in East Nusa Tenggara, Indonesia tend to increase overtime.

Drought characteristics are different among regions depending on the local meteorological and hydrological situation. Drought severity is measured not only by its duration and magnitude, but also by the impact caused. This makes the drought severity level more difficult to be identified and quantified (Wilhite et al., 2000). Resilience to drought becomes one of the main issues to minimise the negative impact of drought. Drought is a climate event that significantly affects ecosystems, livelihoods, and the socioeconomic development of a region. Drought impact on livelihood has received much attention because it has many effects on other sectors, including the socioeconomic aspect. Many authors have focused on assessing drought's impact on livelihood, including Keshavaz et al. (2017), Khayyati and Aazami (2016), Martin et al. (2016), and many others. Drought resilience livelihood has been a big concern in drought risk management. A study by Ranjan and Athaliye (2009) that focused on building drought resilience in agriculture suggested that adoption of water-efficient technology has to be a policy to build resilience to severe and sustained drought. Singh and Chudasama (2016) documented the drought livelihood pathway in India and found that drought resilience livelihoods highly depend on people's perception. Quandt (2018) demonstrated that perceptions of household livelihood resilience vary depending on demographic characteristics, particularly gender and ethnicity*.*

Numerous studies around the world have been conducted to investigate people's perception of drought and its impact. Jarawura (2014) studied the perception of drought among rural farmers in the Savelugu district in the northern Savannah of Ghana. Their paper argued that effective adaptation to drought rests on the integration of both scientific notions and local perceptions of drought by farmers. Dessaie and Sims (2010) focused their study on people's perceptions of drought and climate change, and they found that perception is an important factor for sustainable water management by pointing to barriers to behavioural change. Udmale et al. (2010) studied farmers' perception of drought impact in Maharashtra State, India. The study examined socioeconomic and environmental impacts, adaptation strategies, and opinions on government drought mitigation measures. A recent study by Menghistu et al. (2018) assessed farmers' perception of drought and its impact in Tigray. It found that farmers have a good perception of the severity of drought impact, but the preparadness to deal with its impact was minimal. This condition leads to significant losses in farm income in the livestock sector. Other studies have been conducted on drought perception in other countries, such as the studies of Habiba et al. (2010), Ashraf and Routray (2013), Bahta et al. (2016). Unfortunately, few studies have been conducted regarding drought events in Indonesia, even though several regions in Indonesia have experienced severe drought.

This paper presents the results of a cross-sectional study in East Nusa Tenggara, Indonesia, as one of the regions affected by severe drought for more than a decade. It investigates the perception of weather and seasonal drought forecasts and its impact on livelihood. The perception in this case refers to the adoption to the forecast technology leading to the usage the forecast product. It is part of a broader study aimed to assess households' perception of drought and its impact as well as their adaptation and mitigation strategies. Seasonal drought forecasts are an essential component of an early drought prediction system that can provide advanced warning and alleviate drought impacts (Pozzi et al., 2013). The use of seasonal forecasts is mainly dependent on the actual predictability of drought conditions, which are dependent on the predictability of precipitation (Gianotti et al., 2013). The adoption of seasonal forecasts as an agricultural technology is influenced by farmers’ perception of the forecasts, as documented by Negatu and Parikh (1999). Other forecast products that have been well developed in the agricultural sector to support drought management are crop forecasting (Basso and Liu, 2018; Martins et al., 2018) and seasonal climate forecasting for agricultural producers (Klemm and McPherson, 2017).

The relation between weather and climate forecasts and livelihood has been invesigated by Nidumolu et al. (2018). Their study was conducted in Southern India to link the climate forecasts to rural livelihoods by exploring ways of overcoming barriers, such as those limiting access to information and effective communication of probabilistic forecast information. Solid linkages among farmers and other decision-makers, agricultural scientists, climate scientists, economists, social scientists, and policymakers are required to formulate useful strategies to better manage climate risk. Shiferaw et al. (2014) pointed out that promising technology, including climate forecasting and an early warning system, is essential for effective drought risk management in South Africa to protect livelihood. Roudier et al. (2016) performed a more comprehensive study by assessing the impact of short-term forecasts and seasonal forecasts on Niger millet growers' cropping practices and income. They found that 10-day forecasts alone or a combination of 10-day and seasonal forecasts could be quite beneficial for all types of farmers, and in most cases farmers with access to fertilisers and larger arable land benefit more from forecasts. In line with this, Taylor et al. (2018) pointed out the importance of effective forecasts and warning systems as well as communicating the weather forecast information for better support in decision-making. Considering the essential impact of weather and seasonal drought forecasts on livelihood, this paper assessed the household perception of forecasts and its impact on livelihood in East Nusa Tenggara, Indonesia. The households in East Nusa Tenggara have unique characteristics, where only few percentage of them used the forecast to support the livelihood management. The study is focused on investigating the households characteristics related to their perception on the forecast. Moreover, the impact of using forecasts and responses to forecasts to the loss of livestock and crops is also discussed. A statistical test is applied to determine differences in the livestock lost between households that used forecasts and those that did not, as well as among different types of responses to drought.

## 2 Methodology

The methodology in this paper consists of the description of the study area, data collection, and data analysis. Our research included experimentation on human subjects. This study was approved by the ITS Research Ethics Committee. Therefore, the study was conducted according to and complies with all regulations established in the ethical guidelines by the ITS Research Ethics Committee in the “code of ethics”. All participants provided written informed consent.

### 2.1 Study area

**Fig. 1:**
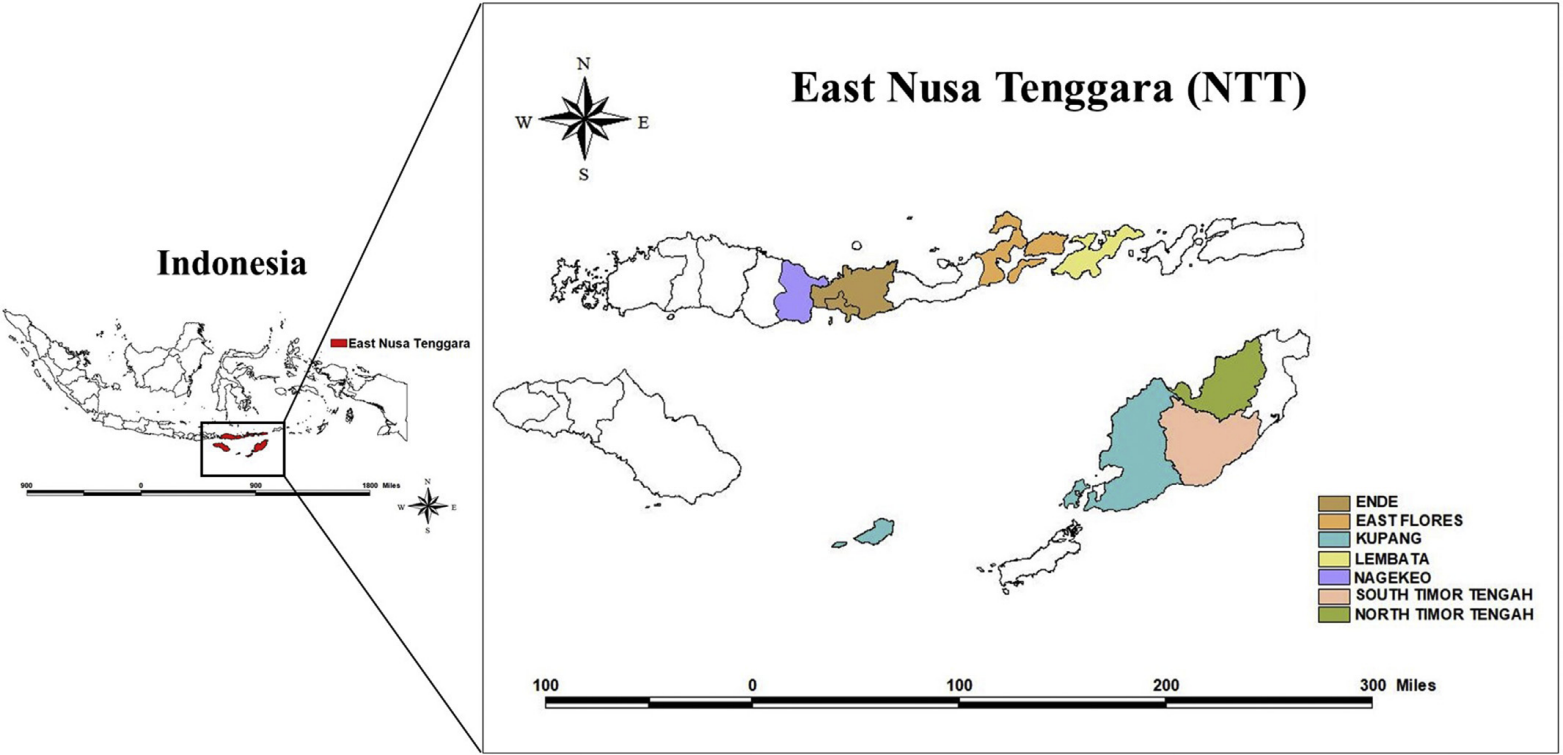
Geographical position of East Nusa Tenggara in Indonesia.

The data are part of the household survey dataset collected in 2018 from seven districs in East Nusa Tenggara (hereafter is called as NTT), Indonesia: Ende, East Flores, Kupang, Lembata, Nagekeo, South Timor Tengah, and North Timor Tengah. See Fig. 1 for the geographical position. The study districts were selected purposively based on their previous history of drought impacts in East Nusa Tenggara. The East Nusa Tenggara region consists of several islands dominated by high-altitude land with a very dry climate condition. East Nusa Tenggara has very short wet periods. During the dry season, East Nusa Tenggara is very dry for long periods (about eight months). The average number of rainy days ranges from 44 to 61 days per year, and the average maximum temperature is 33.2° Celcius. Drought in East Nusa Tenggara is induced by its geographical position, which is located near Australia, where only little water evaporation is transported by the wind from Asia and the Pacific Ocean, resulting in low rain intensity.

The drought in NTT is strongly affected by global phenomenon El Niño, which thus exacerbated the vulnerability of the community. The situation is even worse because the community (especially in rural area) is unable to access reliable water due to improper service of basic facilities such as water and electricity. NTT has also been listed as the top priorty and most vulnerable region in Indonesia to drought. The National Board on Disaster Managemegt (BNPB) reported that in 2017, a total of 11 districts in East Nusa Tenggara (NTT) have been hit by severe dry season. This situation has impacted 640,048 population in 127,940 households, and has been claimed as the worst drought condition within last 5 years. The majority of the population in NTT work as farmers, and hence the water shortage caused by drought has significantly impacted the livelihood and introduced difficulties in agriculture as well as access to clean water leading to harvest failure, food shortage and income reduction. The farmers responded to income reduction by reducing their food expenditure. The geological conditions of the soils in NTT make it difficult for the society to build a drilled well. Consequently, most of them buy clean water through trucks and store a water supply in reservoirs (Aris et al., 2016). To support the livelihood management in NTT, the government through the Agency for Meteorology, Climatology and Geophysics (BMKG) issues an updated weather and seasonal drought forecast. The forecast information is available online at www.bmkg.go.id.

### 2.2 Data collection

The data were collected from an administered questionnaire through face-to-face interviews with household heads or household members. Two stages of sampling were conducted, in which 300 households were chosen randomly from the selected districts and surveyed. The questions in the questionnaire were developed by following the work of Shieldrick (2009), with some modifications. The details about the questionnaire as well as complete dataset can be found in the supplementary file of Kuswanto et al. (2019). The key information analysed in this paper is listed in Table 1.

**Table 1:** Key information analysed in this study.

Variable	Question	Scale
Household characteristics	- Gender - Age - Marital status - Level of education of household head - Level of income - Length of stay - House ownership - Keysource of livelihood	Nominal Ordinal Nominal Ordinal Ordinal Ordinal Nominal
Household agricultural activities	- Do you grow crops? - Water source for the crops - Do you own livestock? - Water source for the livestock	Nominal Nominal Nominal Nominal
Knowledge and perception of drought	- Do you understand about drought? - Causes of drought - Effect of drought - Use of weather and seasonal drought forecasts - Source of information on weather and seasonal drought forecasts - How do you know what the weather and seasonal drought may be like during the dry or rainy season? - Response to weather and seasonal drought forecasts - What do you think of the accuracy of weather forecasts so far?	Nominal Nominal Nominal Nominal Nominal Open question Nominal Open question
Drought impact and experience	- Animals sold in the last year - Reason to sell animals - Lost animals due to drought last year - Lost crops due to drought last year - Impact of drought on livelihood	Ratio Nominal Ratio Ratio Nominal

### 2.3 Data analysis

This research uses both qualitative and quantitative information in the analysis. The statistical method is used to analyse the quantitative data, while the qualitative information is used to produce descriptions of situations, behaviour, and system interactions (Casley and Kumar, 1998) to support the findings from analysing quantitative data. The analysis begins by presenting the sociodemographic characteristics of the households. Further analysis is conducted to investigate the variables influencing the usage of forecast as well as to test the significant difference in crop and livestock loss regarding the following variables: usage of weather and seasonal drought forecasts and response to the forecasts. In this case, the impact is measured by the number of livestock and crops lost due to drought events.

The significance of the impact above will be tested by two or k-independent sample tests. In summary, the hypotheses to be tested are as follows:

- There is a significant influence of usage of weather and seasonal drought forecasts on reducing livestock and crop loss.
- There is a significant influence of response to weather and seasonal drought forecasts on reducing crop loss.

Note that the impact of response to weather and seasonal forecasts is examined only by crop loss because the question relates to agricultural activities.

## 3 Results and discussion

### 3.1 Demographic data and household perception of drought

In this study, a total of 300 respondents were interviewed. From these respondents, 61% were male, 87% were married, and 66% were the household head. The average age of the respondents was 45 years old, while the average age of the household head was 49 years old. The majority of the household heads have only a primary school level of education. Meanwhile, 7.3% of household heads had never gone to school, and only three (1%) household heads have a university degree. This shows that most of the household heads in East Nusa Tenggara are low-educated people. The same fact is observed for the wife. Table 2 reveals that majority of the households in East Nusa Tenggara are categorised as poor, where about 70% of households have a monthly income below 500,000 IDR (about 35 USD), and none of them have a monthly income more than 4,000,000 IDR (250 USD). About 91% of the respondents have been living in their current place more than 10 years, and 3.33% have been living there no more than a year. From the table, we see that about 95% live in their own house, which is either a permanent or semi-permanent house.

**Table 2:** Households’ sociodemographic characteristics.

Variable	Category	Count	Percentage
Monthly income	< IDR 500,000	208	70.03%
	IDR 500,000 – IDR 2,000,000	82	27.61%
	IDR 2,000,000 – IDR 4,000,000	7	2.36%
	> IDR 4,000,000	0	0.00%
Length of stay	<1 year	10	3.33%
	1–5 years	7	2.33%
	5–10 years	10	3.33%
	>10 years	273	91.00%
House ownership	Own	286	95.33%
	Rent	1	0.33%
	Others	13	4.33%

Table 3 provides statistics about household perceptions on drought. The respondents of this survey were questioned to gauge their understanding of drought, the causes of drought, the effect of drought, and the utilisation of weather and seasonal drought forecasts. The survey revealed that about 80% of the respondents understood drought, and only about 16% of them do not understand drought. The survey result also revealed that they understand the causes of drought well, since most of them responded with multiple answers (indicating that drought was a result of several natural phenomena). More than 50% of respondents argued that drought has led to drying of water sources, famine, crop failures, and loss of livestock. However, the respondents answered that drought did not increase food prices or decrease livestock prices.

**Table 3:** Summary of statistics on the effect of drought and source of information for weather and seasonal forecasts.

Variable	Category	Count	Percentage
Effect of drought	Drying of water sources	41	13.67%
	Famine	12	4.00%
	Crop failures	68	22.67%
	Loss of livestock	5	1.67%
	Poor health of humans	5	1.67%
	Poor health of animals	1	0.33%
	Increase in food prices	0	0.00%
	Decline in livestock prices	0	0.00%
	Other (specify)	0	0.00%
	Multiple answers	168	56.00%
Source of information about weather and seasonal forecasts	Radio/TV	24	8.00%
	Extension agent	42	14.00%
	Word of mouth	140	46.67%
	Traditional sources	1	0.33%
	Other (specify)	59	19.67%
	Multiple answers	28	9.33%
	Missing (no response)	6	2.00%

**Fig. 2:**
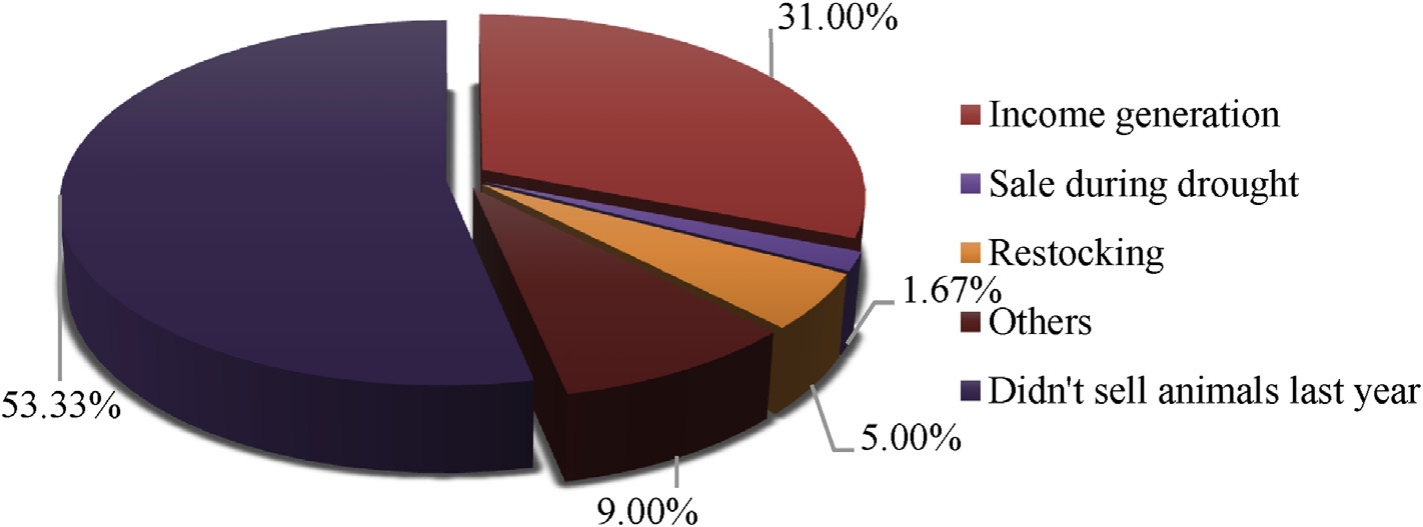
Reasons to sell animals.

**Fig. 3:**
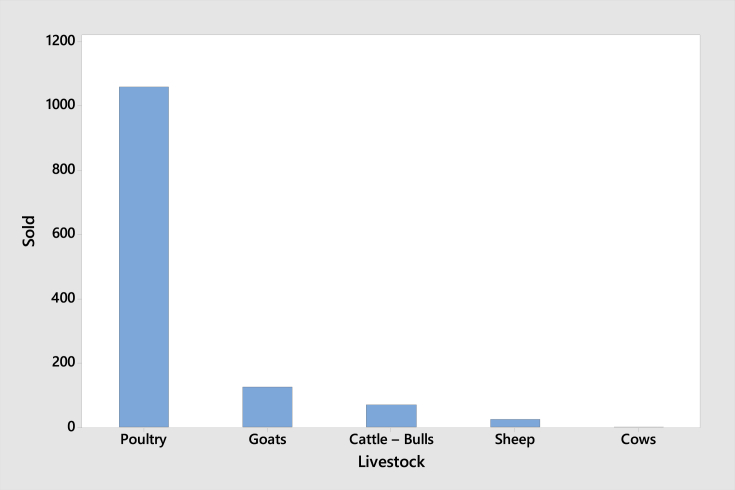
Livestock sold by households due to drought.

Fig. 2 revealed that selling livestock does not really relate to drought, as shown by the small percentage (1.67%) of respondents who sold livestock during drought, and 53.3% respondents did not sell animals. There were about 36% households sold livestock for income generation and restocking. Furthermore, Fig. 3 provides information about the number of livestocks sold as the impact of drought in NTT. The respondents were asked about their experience during the drought the prior year (2017). The majority of the households sold poultry and goats; meanwhile, none of the households had cows.

The survey also recorded the total number of animals lost due to drought in the prior year. There were three types of animals lost: cattle, goats, and poultry. Meanwhile, more variety of crops planted by the households were impacted by drought. Rice, cassava, and maize are three major crops that were negatively impacted by drought.

### 3.2 The forecast usage, response to forecast and its impact on crop and livestock loss

This subsection focuses on discussing the perception of weather and seasonal forecast as well as the survey results about related variables such as level of education of the household head, availability of assets as source of information, key source of livelihood, etc. Again, the perception is defined as the adoption to forecast technology indicated by the usage of forecast. Table 4 provides the percentage of households who used the forecast and not which correspond to the category of each variable.

**Table 4:** Statistics of livelihood management related variables.

Variable	Category	Use forecast		Total
		No	Yes	
Level of education of household head	None	5.9%	0.3%	6.3%
	Primary school	52.6%	12.5%	65.2%
	Secondary school	22.6%	4.9%	27.5%
	Post secondary	3%	0 %	3%
	Total	82.2%	17.8%	100%
Having asset as source of information	Yes	54.7%	15.3%	70%
	No	27.5%	2.4%	30%
	Total	82.2%	17.8%	100%
Key Source of livelihood	Pastoralism	7.3%	0.3%	7.7%
	Agropastoralism	68.3%	15.3%	83.6%
	Small scale business	1.4%	1.4%	2.8%
	Wage employment	5.2%	0.7%	5.9%
	Total	82.2%	17.8%	100%
Grow crops or not	Yes	80.1%	14.6%	94.8%
	No	2.1%	3.1%	5.2%
	Total	82.2%	17.8%	100%
Water source for the crops	Constant supply	3.1%	0.7%	3.8%
	Seasonal	77%	13.9%	90.9%
	Not grow crops	2.1%	3.1%	5.2%
	Total	82.2%	17.8%	100%
Own livestock or not	Yes	73.5%	17.4%	90.9%
	No	8.7%	0.3%	9.1%
	Total	82.2%	17.8%	100%
Water source for the livestock	Constant supply	58.5%	11.8%	70.4%
	Seasonal	15%	5.6%	20.6%
	Not own livestock	8.7%	0.3%	9.1%
	Total	82.2%	17.8%	100%

It is surprising that 82.2% of the households did not use weather and seasonal forecasts to support their daily life especially dealing with livelihood management. The survey revealed that less accuracy of forecast generated by the government (in this case BMKG) became the major reason why they did not use the forecast. Another reason they did not use forecast because they did not know the forecast. As the alternatives of the forecast, the households tend to guess future the weather condition based on their past experience. There are casess where the households did not know at all how the weather may be like during dry or rainy season.

From the table, we see the majority of the household heads are primary school graduate and they did not use forecast. It is very common that people with only primary school education level cannot read well and tends to be illiterate. Therefore, the available forecast information might not be informative enough for them due to low degree of literacy. Furthermore, although 70% of the households have access to forecast information, however, majority of them did not use forecast. It shows that the access to forecast information is not the main reason they did not use forecast. In fact, the government regularly updates the information through public television, radio or website.

Weather and seasonal forecast information is an essential need for agricultural sector, however the survey indicated that only small percentage of households working in agropastoralism used the forecast. Majority of household who grow crops and own livestock also did not use forecast. Moreover, households who need seasonal water source also did not use forecast. Those means that the use of forecast was not driven by whether the forecast is important or not for their livelihood. This findings led to the hypothesis that the use of forecast is more likely to be influenced by the perception about the forecast itself especially the accuracy as well as the clarity of the forecast information, as mentioned by most of the respondents.

From the point of view of the households characteristics, further analysis using Random Forest method of Breiman (2001) can be used to investigate the importance factors influencing the decision to use the forecast. Random Forest has been proven to be a powerful machine learning method compared to others (Kuswanto and Naufal, 2019). The method confirmed that that the usage of the forecast is mainly influenced by the age of the household head. The water source for the crops, whether the household grows crop or not, level of education of household head, ownership of assets as source of information, keysource of livelihood and water source for the crops are important variables, however the degree of the importance is medium. Meanwhile, the influence of length of stay, sex of th household head and ownership of livestock are minor. The examined variables as well as the values of node purity indicating the importance of the variable can be seen in Table 5.

**Table 5:** Node purity index for importance variable.

Variable	Purity Index
Age of the household head	7.5077
Water source for the crops	2.2978
Grow crops or not	1.6982
Level of education of the household head	1.6520
Assets as source of information	1.5626
Key source of livelihood	1.4731
Water source for the livestock	1.4231
Lenght of stay	0.6706
Sex of the household	0.4591
Own livestock or not	0.4186

The indirect impact of the usage of forecast on crop and livestock losses are assessed by testing the significant different of losses between households used forecast and households did not use forecast. The statistical test used to test the sample mean difference is ANOVA test and/or Mann-Whitney test (Mann and Whitney, 1947), while the impact of response to forecast on reducing the crop loss is tested by Kruskal-Wallis as we test more than two categories. The variance homogeneity test is applied prior to testing the sample mean difference (Levene, 1960). Table 6 provides the results of the Levene test applied to three different perception variables for each type of crop.

**Table 6:** Levene test for crop loss.

Crop loss	Use of forecasts		Response to forecasts	
	Levene statistic	P-value	Levene statistic	P-value
Maize	3.265	0.072	9.796	0.000
Cassava	1.940	0.165	9.181	0.000
Sweet potato	0.305	0.581	0.549	0.649
Rice	0.104	0.747	2.245	0.083
Ground nuts	0.164	0.686	4.459	0.004
Beans	1.080	0.300	3.028	0.030
Total crops	0.058	0.810	1.902	0.129

From the table, we reject the null hypothesis if the p-value is less than the significance level (α=0.05). The p-value greater than 0.05 for the case of using weather and seasonal drought forecasts indicates that the variances of all crops are equal. Different results are observed for the response to forecasts, in which only the variances of sweet potato, rice, and total crops are equal. As only two cases (sweet potato and total crops) have equal variance, in order to simplify the analysis, the significant impact of the response to forecast variables to crop loss will be tested with the Kruskal-Wallis test. This test remains valid to be applied to equally variance crop variables. Meanwhile, ANOVA will be applied to the use of forecasts variable.

Table 7 provides the mean of crops loss for households using forecast and not using forecast as the results of applying ANOVA to test the impact of forecast usage on crop losses. It is interesting to note that households using forecast tend to expereince higher loss than households who did not use forecast. However, the p-values of all crop varieties are greater than 0.05, which means that statistically there is no difference in crop loss between households that use forecasts and households that do not. In other words, we conclude that the use of weather and seasonal drought forecasts did not have a significant direct impact on crop loss.

**Table 7:** ANOVA for testing the impact of use of forecasts on crop loss.

Use forecast	Maize	Cassava	Sweet potato	Rice	Ground nuts	Beans	Total crops
Yes	102.843	78.882	5.098	208.039	3.196	22.902	420.960
No	79.149	53.542	6.255	173.153	3.858	13.854	329.813
T-test	1.164	1.261	-0.333	0.381	-0.157	0.616	0.957
P-value	0.245	0.208	0.740	0.703	0.875	0.538	0.339

It is also worth noting that the use of forecasts in this case can be seen as a passive or minimal action. People may use weather forecasts, but whether the information in the forecast is useful depends on further actions. Therefore, investigating the response to drought forecasts is important in this case to provide clear guidance to policymakers about the level of actions that should be carried out by households to minimise the drought impact.

The crop loss is more likely to be directly impacted by the response to forecast. Table 8 provides the results of applying the Kruskal Wallis test to know the impact of the response to forecasts on crop loss, for the households who use forecast. We see that crop (maize, rice, beans, and the total crops) loss was significantly impacted by the response to forecasts, shown by the p-value less than 0.05. However, the impact is different for each crop. There was no significant impact on cassava, ground nuts, or sweet potato. This finding is consistent with the findings of studies conducted by Uya et al. (2015), and others. These studies found that the productivity of maize and rice is highly impacted by drought. Lunduka et al. (2017) therefore suggested that drought-tolerant maize varieties be adopted by farmers to minimise loss.

**Table 8:** Kruskal-Wallis test for testing response to forecasts on crop loss.

Response to forecast	Maize	Cassava	Sweet potato	Rice	Ground nuts	Beans	Total crops
Change normal agriculture practice	47.982	23.051	2.758	90.396	7.017	2.000	173.206
Try to get more information	145.843	59.625	8.125	184.781	6.250	0.781	405.406
Didn't do anyting difference	85.696	69.815	6.293	210.064	2.532	22.194	396.597
Chi-square	11.139	8.849	0.484	16.450	4.874	15.889	13.236
P-value	0.025	0.065	0.975	0.002	0.300	0.003	0.010

It indicated that the use of forecasts would not directly lead to crop loss reduction. Farmers might know some information from the forecast; however, they have no capacity to adapt to it. In fact, the planted crops were very vulnerable toward high drought magnitude. A study by Kuswanto et al. (2018) revealed that the drought magnitude (duration and intensity) in East Nusa Tenggara was significantly high. This result is consistent to some extent with the study of Keshavaz et al. (2017), who studied the livelihood vulnerability to drought in Iran. They argued that drought is the main threat to livelihood security, while the interaction between drought intensity and its duration leads to more vulnerability, especially for crops. Meanwhile, planted crops such as maize, cassava, sweet potatoes, ground nuts, and beans are vulnerable to weather and climate conditions. These crops are too vulnerable to drought in East Nusa Tenggara, and hence using the forecast alone did not help to minimise the negative impact of drought.

The survey revealed that people who changed agricultural practice tend to have less average maize loss (47.982) than those who did not do anything differently (85.696) or obtained more information (145.843). The same fact is observed for loss of rice and beans. People who got more information had the relatively highest crop loss. This might be due to the late action caused by too many considerations, similarly to the people who choose to make more than one choice. Meanwhile, different responses did not significantly impact the loss of cassava, sweet potatoes and groung nuts as the p-values for these crops did are greater than 0.05. In summary, we found that the response to forecasts has a significant impact on the total loss of crops. People who changed their agricultural practice had significantly lower crop loss than people who tried to obtain more information or did not do anything differently. These findings show that the perception about the importance of weather and seasonal drought forecasts is essential to minimise the negative impact of drought.

For animal loss, we only investigate the impact on cattle bulls, goats, poultry, and the total livestock. The Levene test of homogeneity in variance shows that the variance of cattle bulls is equal, but it is not equal for goats, poultry, and the total loss. These results lead to the choice of using the Mann–Whitney U test to test the impact of using forecasts.

The results of the Mann–Whitney U test shown in Table 9 reveal that the use of forecasts significantly reduced the loss of goat and poultry, but not cattle bulls. Cattle bulls are strong enough to adapt to the drought situation, unlike poulty and goats, where households using forecasts experienced lower loss than households that did not use forecast.

**Table 9:** Levene test and Mann–Whitney U test for livestock loss.

	Homogeneity variance test		Mean difference test	
	Levene statistic	P-value	Mann–Whitney U	P-value
Cattle bulls	3.133	0.078	6035.5	0.185
Goats	54.608	0.000	5603.5	0.001
Poultry	4.159	0.042	5707.5	0.002
Total animals	7.155	0.008	5270.5	0.001

## 4 Conclusions

Weather and seasonal forecast is an important aspect to minimize drought risk, especially to deal with the livelihood (crop and livestock) management. This study revealed very low percentage of households in NTT who used forecast information. One of the reasons not to use forecast is about the low accuracy of the forecast information provided by the government. Moreover, low education backgroud might become a barrier to gather information about weather and seasonal forecast under the current situation. Due to its importance, households should receive a valid and reliable forecast. This will influence the willingness to use forecasts in the future. Effective comminication to households, as pointed out by Taylor et al. (2018), can be implemented. In fact, our survey revealed that majority of the respondents said that they did not believe the forecasts issued by the government due to inaccuracy. Therefore, the goverment must improve forecast accuracy. One of the possible ways to improve the forecast accuracy could be to integrate the use of Numerical Weather Prediction (NWP) outputs in generating the forecast (short term forecast, sub-seasonal and seasonal forecast). The NWP technology has been proven to be able to generate more reliable and accurate forecasts in many countries. Considering the socio-characteristics of the households in East Nusa Tenggara, the forecast should be presented in a simple and interesting way or platform. A tool has to be designed so that it can be easily used by low-educated people. An effective strategy needs to be considered to deliver the information to remote areas.

The present study is the first in Indonesia to investigate the relationship between the use of weather and seasonal drought forecasts and crop and livestock losses, particularly in Indonesia. The present paper analysed the impact of the usage of weather and seasonal drought forecasts as well as the response to the forecasts on crop and livestock losses. The data were subjected to a non-parametric tests during the analysis, the Mann–Whitney U test and Kruskal-Wallis test. The findings of this study can provide guidance to policymakers for a better mitigation strategy to increase community awareness to use weather and seasonal drought forecasts. The results of this study showed that the use of weather and seasonal forecasts does not have a significant direct impact on crop loss, however, this does not mean that using forecast is not important. Indirect impact in this case means that in order to reduce the crop loss, it is not enough for the farmers to only use the forecast information. Further action carried out by the farmers after getting information about forecast is more important to minimise the negative impact of drought. The final analysis investigated the importance of response type to crop loss. The results showed that the response choice has a significant impact on crop loss especially maize, rice, and beans. Those who adapt to drought by responding to forecasts by changing agricultural practices experienced less crop loss than those who did not do anything differently or obtained more information. Meanwhile, the losses of cassava, sweet potato, and ground nuts were not impacted by the choice of response. This study also showed that the use of weather and seasonal drought forecasts does not impact cattle bull loss in East Nusa Tenggara, Indonesia. Meanwhile, goats and poultry are significantly impacted by the use of weather and seasonal forecasts.

From the perspective of policy implications, the findings of this study are important and informative. They show that intervention to increase the awareness of using weather and seasonal drought forecast information is important in order to minimise the impact of drought, especially for crop management. Furthermore, adaptation to drought by changing agricultural practices after getting forecast information is important to minimise loss. One strategy is to plant drought-tolerant crop varieties, as suggested by Lunduka et al. (2017), or to practice smart climate agriculture, as has been well discussed by Lipper et al. (2014). Capacity building on this issue is required.

## Declarations

### Author Contribution Statement

H. Kuswanto: Conceived and designed the experiments; Performed the experiments; Analyzed and interpreted the data; Contributed reagents, materials, analysis tools or data; Wrote the paper.

F. Hibatullah: Conceived and designed the experiments; Performed the experiments; Contributed reagents, materials, analysis tools or data.

E. S. Soedjono: Conceived and designed the experiments; Contributed reagents, materials, analysis tools or data; Wrote the paper.

### Funding Statement

This work was supported by the National Academy of Science (NAS)-USA and USAID through the Partnerships for Enhanced Engagement in Research (PEER) research grant and partially supported by the Ministry of Research, Technology and Higher Education Indonesia through the National Strategic Research Scheme and the Massachusetts Institute of Technology-Indonesia Research Alliance (MIRA) program.

### Competing Interest Statement

The authors declare no conflict of interest.

### Additional Information

No additional information is available for this paper.

## Appendix C Supplementary data

Final Questionnaire_for submission Final Questionnaire_for submission
